# Effect of Travel Expenditure on Life Satisfaction for Middle-Aged and Older Adults in Korea: Moderating Effect of COVID-19 Pandemic

**DOI:** 10.3390/ijerph192013523

**Published:** 2022-10-19

**Authors:** Joonho Moon, Won Seok Lee, Jimin Shim

**Affiliations:** 1 Department of Tourism Administration, Kangwon National University, Chuncheon 24341, Korea; 2 Department of Tourism and Recreation, Kyonggi University, Seoul 03746, Korea

**Keywords:** life satisfaction, middle-aged and older Koreans, travel expenditure, inverted U shape, COVID-19

## Abstract

The aim of this research was to examine the effects of travel expenditures on life satisfaction in the context of the COVID-19 pandemic. For the research, a curvilinear relationship was established between life satisfaction and travel expenditures that was then compared between 2018 and 2020. The study subjects were middle-aged and older adults who completed the 2018 and 2020 waves of the Korean Longitudinal Study of Aging. Demographic, travel expenditure, and life satisfaction data were collected, and quadratic regression analysis was conducted to examine the effects of travel expenditures on life satisfaction before and during the COVID-19 pandemic. The first-order condition was computed to determine the optimal level of travel expenditures to maximize life satisfaction, and the results exhibit that the utility of travel expenditure decreased during the COVID-19 pandemic.

## 1. Introduction

Korean society is aging steadily if not rapidly. According to Statistics Korea [1], which has the most updated statistics, the median age in numerous areas of Korean society is forecasted as being over 60 years old, with aging accelerated by low national birth and fertility rates. In this situation, understanding behavioral patterns among middle-aged and older citizens will enable the building of more adequate policies for addressing the needs of this aging population, and effective policy measures for welfare will improve and maintain quality of life. Hence, it is valuable to inspect the characteristics of the older population in Korea to assess current and potential needs. Previous researchers asserted that life satisfaction is an indicator of individual happiness [2,3], which led subsequent scholars to investigate the determinants of life satisfaction [4,5,6,7,8]. The fertile evidence suggests that life satisfaction is worth investigating, and therefore, this research adopted life satisfaction as the main explained variable.

The first domain of this research was travel expenditures. Researchers have established travel as an essential element in life satisfaction [4,9], and taking advantage of nearly all goods, services, and resources requires payment. Indeed, researchers have determined cost to be a crucial element in understanding individuals’ behavior patterns. However, despite the importance of integrating both elements, scholars have scantly explored the effects of travel expenditures on life satisfaction. To attempt to address this research gap, this work elected to study travel expenditures as the main explanatory attribute of life satisfaction among middle-aged and older Korean adults. 

The second area of this research is the Corona Virus Disease 2019 (COVID-19) pandemic, which not only changed daily life patterns globally but also heavily impacted global economic conditions [10,11,12,13]. Specifically, markets during the COVID-19 pandemic have valued noncontact consumption [14,15,16]. Unfortunately, travel-related businesses are built on contact and thus were tremendously damaged by the COVID-19 pandemic. Meanwhile, older adults comprise a large part of the travel consumer population, so the COVID-19 pandemic notably changed their travel consumption characteristics. 

In total, the purpose of the current work was to investigate the effects of travel expenditures on the life satisfaction of middle-aged and older Koreans considering the COVID-19 pandemic as a moderating variable. Theoretically, this research sheds light on the literature by elucidating the association between travel expenditure and life satisfaction. Additionally, this research is valuable in that it identified the effect of the COVID-19 pandemic on the relation between travel expenditure and life satisfaction as longitudinal manners. Practically, the aim of this research is to offer information for establishing more appropriate resource allocation and designing government policies regarding travel expenditures. Since infectious diseases such as COVID-19 could be repeated, it is worthwhile scrutinizing the behavioral pattern during the COVID-19 pandemic. By doing so, the policy makers could reference this information to build more adequate policy during the COVID-19 pandemic. 

## 2. Review of Literature and Hypotheses Development

### 2.1. Life Satisfaction

Life satisfaction refers to an individual’s appraisal of his or her own life [4,6], and scholars claimed that life satisfaction is a sort of attitude towards life rather than temporary emotion [6,8]. Researchers have frequently highlighted life satisfaction as their main study attribute. For instance, Clair et al. [17] demonstrated the effects of isolation on life satisfaction, and Chen et al. [4] used life satisfaction as a dependent variable to examine gender differences. Khodabakhsh [5] investigated the determinants of life satisfaction using older adults as survey participants, and Tavares [18] tested the impacts of health on life satisfaction in the context of Portuguese older adults. Wettstein et al. [7] also explored the influence of COVID-19 on older adults’ life satisfaction. Life satisfaction has been widely studied as an outcome variable in the literature. Moreover, previous studies stated that life satisfaction is influenced by multiple elements, and it is valuable to investigate significant attributes to account for life satisfaction [7,8,18]. 

### 2.2. Travel Expenditure

Travel expenditures are the costs related to traveling activities [19,20]. Both Chen et al. [21] and Campón-Cerro et al. [22] reported that tourism played an important role in enhancing life satisfaction, and Mahadevan and Fan [9] found the same impact specifically among older adults. However, scholars have rarely examined the associations between travel expenditures in particular and life satisfaction. Therefore, it is essential to attest the impact of travel expenditure on life satisfaction because there might be a big difference between travel itself and travel cost. Travel expenditures primarily include costs for transportation, lodging, and food but encompass other expenses as well [19,23,24]. In general, excessive costs decrease the utility of goods [25,26], and the law of diminished marginal utility contends that repeated consumption of certain goods can decrease their value as well [27,28]. In the context of this study, repeated travel causes higher expenditures, which should be likely to decrease the effect of traveling on life satisfaction. Therefore, this study proposes the following research hypothesis:

**H1.**  *There is an inverted-U-shaped association between travel expenditures and life satisfaction*.

### 2.3. Impact of COVID-19

The COVID-19 pandemic radically changed societies worldwide, mainly in the form of significant isolation [29,30], and severely curtailed personal contact [31,32]. Because COVID-19 is so infectious, individuals isolated because they valued their safety [15,33], and this of course changed people’s travel consumption patterns. In detail, international travel declined, and individuals focused on domestic travel that allowed for maintaining distances from others because travel requires personal contact with many people [10,14,16]. Moreover, consumption of services became conservative to minimize risky contacts with service providers [12,15]. Travel-related businesses have been severely affected by the COVID-19 pandemic because travel is a service-intensive industry which requires numerous instances of personal contact for product consumption [12,15,33]. Hence, COVID-19 made such dramatic changes to consumption patterns in general and to travel consumption. Therefore, it is anticipated that the utility of travel is likely to appear variedly during the COVID-19 pandemic as compared to before the COVID-19 pandemic. Thus, this research also proposed the following research hypothesis:

**H2.**  *There is a significant difference between “pre-COVID 19” and “post-COVID-19” in the relationship between travel expenditures and life satisfaction*.

## 3. Method

### 3.1. Research Model and Data Collection

**Figure 1:**
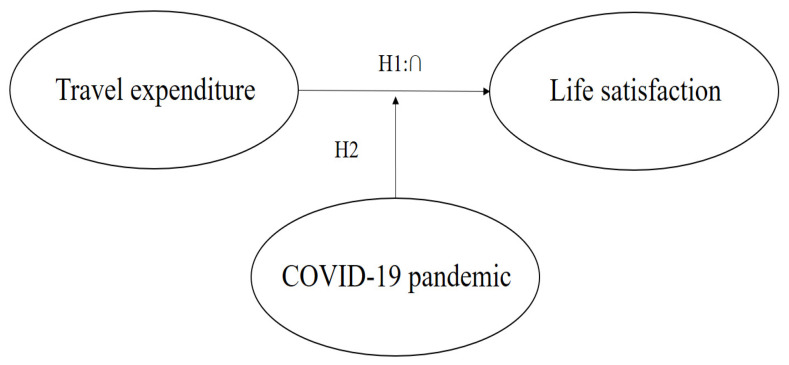
Research model.

Figure 1 presents the study research model. The independent variable of this work was travel expenditures, and the dependent variable was life satisfaction. This work identified a curvilinear (inverted U) effect of travel expenditures on life satisfaction and proposed that the impacts of travel expenditures on life satisfaction differed significantly between before and after COVID-19.

This study obtained the data for this study from an archival source, specifically the Korean Longitudinal Study of Aging administered every two years by the Korea Employment Information Service. Because the data were derived from a secondary data set, this work executed data cleaning in the cases of both missing information and refusal to respond the survey questions. For this study, the survey data collected were from the 2018 and 2020 survey waves, which encompassed the first year of the COVID-19 pandemic. There were 2928 observations in 2018, which declined to 2187 after the data cleaning, and it can be inferred that there were fewer survey participants after the onset of COVID-19.

### 3.2. Illustration of Variables and Data Analysis

The dependent variable in this research was life satisfaction (LSA), which the survey respondents rated on a scale from 0 to 100. Respondents rated their travel expenditures (TRE) as annual amounts in units of KRW 10,000. COVID-19 was the study dummy variable (0 = pre-COVID-19, 1 = post-COVID-19), and there were three control variables: gender (GEN), age (AGE), and personal assets (AST), where GEN was binary (0 = male, 1 = female), AGE was each respondent’s physical age, and AST was also rated in units of KRW 10,000. Table 1 presents the statistics for the variables.

For the data analysis, this study computed the means and standard deviations for all variables in both years, 2018 and 2020; this work then calculated a correlation matrix of the two years. This study adopted ordinary least square regression analysis because COV variable could control time effect as a sort of fixed effect model, which incorporates dummy variable to control year effect [34,35]. This research also carried out quadratic regression analysis to test the curvilinear effect of TRE on LSA. In quadratic regression analysis, the model incorporates squared independent variables into the regression equation, which enables researchers to compute optimal point using differentiation [34,36]. After this work calculated the coefficients in the quadratic regression model, this research performed differentiation to reach the first-order condition that enabled us to calculate the optimal TRE for maximizing LSA [34,36]. To test for significant differences between pre- and post-COVID-19, this study incorporated into the regression model both COV and two interaction variables: TRE^2^ × COV and TRE × COV. The following is the regression equation for this research:

*LSA_it_* = *β*_0_ + *β*_1_ + *TRE_it_*^2^ + *β*_2_*TRE_it_* + *β*_3_*COV_it_* + *β*_4_*TRE*^2^ × *COV_it_* + *β*_5_*TRE* × *COV_it_* + *β*_6_*GEN_it_* + *β*_7_*AGE_it_* + *β*_8_*AST_it_* + *e_it_* (1)

where *e* is residual, LSA is life satisfaction, TRE is travel expenditure, COV is the COVID-19 pandemic, GEN denotes gender, AGE is survey participants’ physical age, and AST is personal assets.

## 4. Results

### 4.1. Descriptive Statistics and Correlation Matrix

The mean LSAs in 2018 and 2020 were 62.33 and 63.47, respectively. TRE in 2018 was 70.80, and its standard deviation (SD) was 136.19, and TRE in 2020 was 73.32 with an SD of 167.50. Table 2 presents the statistics for the other variables in both years. 

Table 3 is the correlation matrix. In 2018, LSA positively correlates with TRE (r = 0.207) and AST (r = 0.170), while LSA negatively correlates with GEN (r = −0.069) and AGE (r = −0.236). In 2020, LSA also positively correlates with TRE (r = 0.122) and AST (r = 0.170), whereas LSA negatively correlates with GEN (r = −0.047) and AGE (r = −0.183). TRE negatively correlates with AGE in both 2018 (r = −0.182) and 2020 (r = −0.107). In 2018, AST positively correlates with TRE (r = 0.268) and negatively correlates with GEN (r = −0.150) and AGE (r = −0.064). In 2020, AST positively correlates with TRE (r= 0.293), but AST negatively correlates with GEN (r = −0.162) and AGE (r = −0.067).

### 4.2. Results of Hypothesis Testing

Table 4 shows the results of the multiple linear regression analysis. Model 1 and Model 2 reflect 2018 and 2020, respectively, and Model 3 is the regression analysis of the interaction effect of COV. All three models are statistically significant given the F values (*p* < 0.05). In both years, TRE^2^ and TRE exerted significant effects on LSA: 2018, β = −1.4 × 10^−5^ and β = −0.029 (both *p* < 0.05); 2020, β = −3.6 × 10^−5^ (*p* < 0.1) and β = −0.011 (*p* < 0.05), both, respectively. Based on the first-order condition computation, the optimal TRE for maximum LSA in 2018 was 1008.23, and in 2020, the optimum was 1570.13. In both years, AGE had negative effects, and AST had a positive influence. The results for Model 3 showed that the coefficients of TRE^2^ × COV (β = 1.1 × 10^−5^, *p* < 0.05) and TRE × COV (β = −0.186, *p* < 0.05) were significant, indicating significantly different curvilinear relationships for the effects of travel expenditures on life satisfaction among older Korean adults between before and after COVID-19. In sum, H1 and H2 are supported. 

## 5. Discussion

The purpose of this work was to research the effects of travel expenditures on life satisfaction among middle-aged and older adults in Korea. Another purpose of this research was to investigate the differences in that relationship between before and after the COVID-19 pandemic using data from the 2018 and 2020 waves of the Korean Longitudinal Study of Aging. This study proposed that the impacts of travel expenditures on life satisfaction would be different in the two periods, and the results revealed a curvilinear (inverted U shape) impact of travel expenditure on life satisfaction that was indeed different in the two years. The results are varied with Chen et al. [4] and Campón-Cerro et al. [22] in that tourism itself exerted a linear effect on life satisfaction, while this work disclosed the curvilinear association between travel expenditure and life satisfaction. 

Specifically, for both years, this work calculated the optimal travel expenditures for maximizing life satisfaction and found the 2018 amount to be 10,082,300 KRW. However, the optimal amount in 2020 was 15,701,300 KRW, indicating that gaining satisfaction from travel in the COVID-19 era requires that middle-aged and older Koreans spend more on their travel. Moreover, the magnitude of correlation coefficient between life satisfaction and travel expenditure is stronger in the case of pre-COVID-19 as compared to post-COVID-19. It can be inferred that the senior citizens might need to spend more their money on travel to attain the same utility post-COVID-19. This could reflect that the COVID-19 pandemic had so negatively affected moods that it took more money to recover, reflecting a social cost of the pandemic. However, it is also the case that owing to necessary changes in service delivery and other disruptions, many aspects of travel became more expensive to provide, so any travelers would necessarily pay more. The findings of this work were aligned with the argument of extant literature in terms of changed behavioral pattern during COVID-19 because it is found that middle-aged and older adults perceived travel expenditure during the COVID-19 [29,30,31,32]. In addition, the correlation coefficient between travel expenditure and personal assets was stronger in the case of post-COVID-19. It implies that the economic condition caused wider gaps for travel during the COVID-19 pandemic; it ultimately might affect the overall quality of life.

Regarding the dummy variable results, life satisfaction improved during the period. A possible explanation for this finding is that conditions worsened during the pandemic for participants with poor life satisfaction such that they could not continue to take part in the survey by 2020. Indeed, the descriptive statistics reflect fewer observations in 2020 than in 2018, which could also have reflected attrition from COVID-19 illness and deaths.

## 6. Conclusions

### 6.1. Theoretical and Practical Implications

This study contributes to the literature in several ways. First, this study identified an inverted-U-shaped relationship between travel expenditures and life satisfaction, which no other researchers have explored. This work shed light on the relationship and demonstrated the law of diminishing marginal utility in the association between travel expenditures and life satisfaction using middle-aged and older Korean adults as the study subjects. Therefore, this study found the curvilinear effect of travel expenditure on life satisfaction, whereas previous research limited to the linear association between life satisfaction and travel itself [4,22]. It demonstrates the difference between travel itself and travel expenditure to account for life satisfaction. Next, this work contributed to the identification of the differing curvilinear relationships between travel expenditures and life satisfaction between before and after the COVID-19 pandemic by studying the data of respondents to the 2018 and 2020 waves of a national survey. This work calculated the optimal travel expenditures for maximum life satisfaction and found a considerably higher optimal amount following the outbreak of the COVID-19 pandemic.

This study has policy implications in addition to literature contributions. First, policy makers might consider travel subsidies for older Korean adults given the positive impacts of travel on life satisfaction and the higher post-COVID-19 travel costs; subsidies could offset the main travel expenses: transportation, lodging, and food. Conversely, policy makers could offer subsidies to travel-related businesses that would allow providers to lower their prices, which in turn would result in better utility for travelers. Moreover, policy efforts should likely target poor older adults because they are unlikely to allocate resources to traveling, even though making such allocations according to economic class might be complex and controversial. In addition, policy makers might need to focus more on older female adults because the results showed lower life satisfaction among older women.

### 6.2. Limitations and Suggestion for Future Research

This study does have limitations. First, this study used only a single item to measure life satisfaction, the Korean Longitudinal Study of Aging’s 0–100 scale; future researchers could consider multiple measures of life satisfaction. Moreover, this research was limited to the Korean case, and results could be more robust from a more diverse geographic sample.

## Figures and Tables

**Table 1:** Variable description.

Name	Code	Description (Unit)
Life satisfaction	LSA	(0 = Very poor, 100 = Very good)
Travel expenditure	TRE	Annual travel expenditure (unit: 10,000 KRW)
COVID-19 pandemic	COV	(0 = pre-COVID-19, 1 = post-COVID-19)
Gender	GEN	(0 = Male, 1 = Female)
Age	AGE	Physical age of survey participants
Personal assets	AST	Personal assets (10,000 KRW)

Note: KRW denotes Korean won.

**Table 2:** Descriptive statistics (N = 2928 in 2018) (N = 2187 in 2020).

Variable	Mean of 2018	SD of 2018	Mean of 2020	SD of 2020
LSA	62.33	16.74	63.47	16.39
TRE	70.8	136.19	73.72	167.5
GEN	0.57	0.49	0.57	0.49
AGE	69.55	10	70.94	9.63
AST	23,660.36	36,384.96	25,758.98	40,133.88

Note: SD denotes standard deviation.

**Table 3:** Correlation matrix.

Variable	1	2	3	4	5
1. LSA	1	0.122 *	−0.047 *	−0.183 *	0.170 *
2. TRE	0.207 *	1	−0.063 *	−0.107 *	0.293 *
3. GEN	−0.069 *	−0.033	1	0.034 *	−0.162 *
4. AGE	−0.236 *	−0.182 *	0.032 *	1	−0.067 *
5. AST	0.170 *	0.268 *	−0.150 *	−0.064 *	1

Note: * *p* < 0.05, lower diagonal is 2018, upper diagonal is 2020.

**Table 4:** Results of hypotheses testing.

Variable	Model1 (2018)	Model2 (2020)	Model3
	β (t-Stat)	β (t-Stat)	β (t-Stat)
Intercept	76.640 (36.35) **	76.494 (29.48) **	75.595 (46.01) **
TRE^2^	−1.4 × 10^−5^ (−4.94) **	−3.6 × 10^−6^ (−1.88) *	−1.4 × 10^−5^ (−5.00) **
TRE	0.029 (8.71) **	0.011 (3.33) **	0.029 (8.94) **
GEN	−0.778 (−1.55)	−1.291 (−2.20) **	−0.996 (−2.62) **
AGE	−0.189 (−6.21) **	−0.147 (−3.96) **	−0.172 (−7.31) **
AST	3.3 × 10^−5^ (5.38) **	3.4 × 10^−5^ (5.51) **	3.3 × 10^−5^ (7.71) **
COV			2.462 (5.29) **
TRE^2^ × COV			1.1 × 10^−5^ (3.24) **
TRE × COV			−0.186 (−4.06) **
F-value	49.66 **	19.84 **	44.65 **
R^2^	0.0783	0.0435	0.0639

Note: Dependent variable: LS, * *p* < 0.1, ** *p* < 0.05, optimal frequency to maximize LS: Δ/ΔTRE of 2018 = 1008.23, Δ/ΔTRE of 2020 = 1570.13.

## Data Availability

Not applicable.
